# Laparoscopic versus open surgery for rectal cancer: a meta-analysis of 3-year follow-up outcomes

**DOI:** 10.1007/s00384-016-2506-9

**Published:** 2016-02-04

**Authors:** Dachuan Zhao, Yibin Li, Senming Wang, Zonghai Huang

**Affiliations:** 1 Department of General Surgery, Zhujiang Hospital, Southern Medical University, Guangzhou, 510282 People’s Republic of China; 2 Department of Oncology, Zhujiang Hospital, Southern Medical University, Guangzhou, 510282 People’s Republic of China

**Keywords:** Rectum cancer, Laparoscopy, Open surgery, Survival rate, Meta-analysis

## Abstract

**purpose:**

We wished to evaluate the effectiveness of laparoscopic and open surgery for patients with rectum cancer through a meta-analysis.

**Methods:**

We searched PubMed, EMBASE, and Cochrane database until June 30, 2015, to identify eligible studies. Randomized controlled trials comparing laparoscopic with open surgery for rectum cancer were included. Meta-analysis was performed using the search strategy following the requirement of the Cochrane Library Handbook. Three-year overall survival (OS) and disease-free survival (DFS) were the main endpoints.

**Results:**

Eight randomized controlled trials comprising 3145 patients matched the selection criteria. Meta-analysis showed no significant difference between laparoscopic and open surgery in 3-year overall survival (OS) and disease-free survival (DFS) (hazard ratio (HR)_3-year OS_ = 0.83, 95 % CI [0.68–1.01]; *P* = 0.06; HR_3-year DFS_ = 0.89, 95 % CI [0.75,1.05]; *P* = 0.16). No evidence of publication bias was observed.

**Conclusion:**

Our meta-analysis supported the notion that based on the 3-year DFS and OS, oncological outcomes are comparable after laparoscopic and open surgery for rectal cancer.

## Introduction

Colorectal cancer is one of the most common malignant tumors worldwide [1]. Its incidence rate in western developed countries has been in the third place among all malignant tumors and the fifth common malignant tumor in China [2–4]. With the improvement of technology, the number of therapeutic modalities available for rectal cancer has increased dramatically.

In recent years, accumulating evidence indicate that laparoscopic surgery was associated with lower blood loss, earlier return of bowel movement, and reduced length of hospital stay as compared to open surgery [5–7].

However, evidence is lacking to evaluate whether the long-time survival after laparoscopic resection of rectal cancer is not inferior to open surgery. As a result, the latest National Comprehensive Cancer Network (NCCN) Clinical Practice Guidelines for Rectal Cancer stated that “Laparoscopic surgery is preferred in the setting of a clinical trial” [8].

Although meta-analysis studying some of these trials had shown equivalence, not all reported long-term data including the Comparison of Open versus laparoscopic surgery for mid or low REctal cancer After Neoadjuvant chemoradiotherapy (COREAN) trial. More recently, the COlorectal cancer Laparoscopic or Open Resection (COLOR II) trial has released 3-year survival rates that can now be included. It is necessary to update the result of meta-analysis.

The aim of this paper was to perform a meta-analysis of 3-year survival rates of the clinical trials to evaluate the effectiveness of laparoscopy and open surgery for rectum cancer.

## Materials and methods

### Search strategy

We searched for relevant studies according to the search strategy of the Cochrane Collaboration [9]. Two of the authors independently completed an online search of PubMed, Embase, and Cochrane library for studies for RCTs that compared laparoscopic with open surgery for rectal cancer. The following search terms were used: “rectum cancer [Mesh]/colorectal neoplasms/colorectal cancer/colorectal tumor/colorectal adenocarcinoma/rectal neoplasms/rectal cancer/rectal tumor/rectal adenocarcinoma/rectum neoplasms/rectum cancer/rectum tumor/rectum adenocarcinoma,” “laparoscopy[Mesh]/laparoscopy/laparoscope/laparoscopic,” and “laparotomy[Mesh]/open surgery/open abdominal surgery/conventional surgery/laparotomy.” No language limitation or other restrictions such as research design was imposed in this search. The search included literature published until April 2015 with no lower date limit. The computer search was supplemented with manual searches for references of included studies. The reference lists of obtained studies were also reviewed to identify relevant citations. The abstracts or full text of all potentially relevant studies were independently scrutinized by two reviewers; any disagreements were resolved and reached a consensus after discussion. All the results were limited by “randomized controlled trials”.

### Inclusion and exclusion criteria of trials

To be eligible for the meta-analysis, the following selection criteria were set: (1) treatment of rectum cancer by laparoscopy versus open surgery, (2) prospective randomized controlled clinical trial, (3) the age of the patient population should be over 18 years, and (4) the description of the details of 3-year overall survival rate (OS) and disease-free survival (DFS) rate.

Studies were excluded if they did not have appropriate data or could not able to extract available data from the published result, and [3] deal with recurrent rectum cancer or metastatic carcinoma.

### Qualitative analysis

The risk of bias in RCTs was assessed following Cochrane recommendations, considering random sequence generation, allocation concealment, blinding of participants and personnel, blinding of outcome assessment, incomplete outcome data, and selective reporting [9]. Each category was assessed as yes (low risk of bias), unclear, or no (high risk of bias), and summarized in a table with plus, question mark, or minus signs, respectively.

Publication bias was evaluated by funnel plots and Egger’s regression [10].

### Statistical analysis

Log hazard ratios and their variance were used as the summary outcome measure from all trials in the meta-analysis. For each trial, hazard ratios (HR) with the 95 % confidence interval (95 % CI) of the survival rate was derived and calculated using either the fixed-effects model or the random-effects model [11]. For each meta-analysis, Cochrane’s Q statistic was first calculated to assess the heterogeneity between studies. For *P* values less than 0.1, the assumption of homogeneity was deemed invalid, and the random effects model was used; otherwise, data were assessed using the fixed-effects model. In addition, a funnel plot was used to examine a potential publication bias. Statistical analysis was independently performed by two statisticians using the software program Review manager (version 5.1).

As Marco Braga’s trial reported Dukes staging, Dukes A, B, and combined C1 and C2 were treated as stages I, II, and III for the purposes of inclusion in the final analysis by stage (American Joint Committee on Cancer).

## Results

### Study identification and quality assessment

**Fig. 1:**
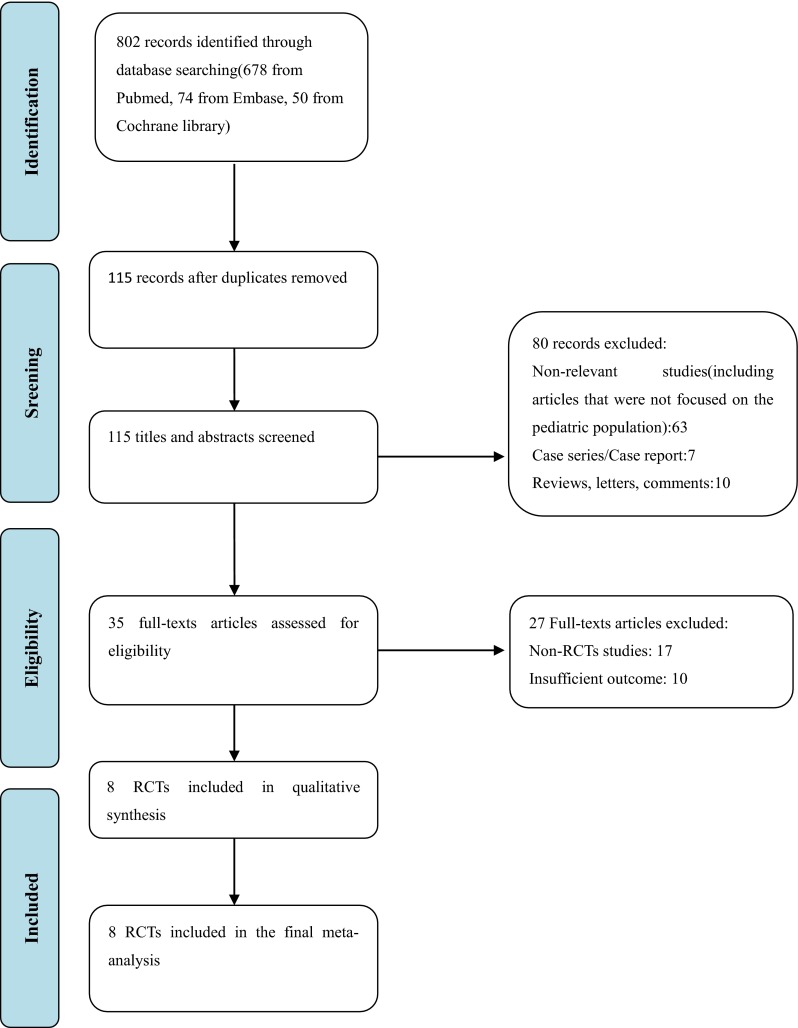
Number of rectum cancer patients included in the meta-analysis

A total of 802 studies were identified by the electronic searches. After excluding duplicates, 115 articles remained, 80 of which were excluded: 63 were not relevant, 7 were case series/case reports, and 10 were reviews/letters/comments. Thirty-five considered articles were reviewed in depth, and a full examination of the text was performed. Seventeen studies were excluded because they were non-RCT studies. 10 studies were excluded because of the insufficient outcome. At last, eight studies involving a total of 3145 rectum cancer patients were included into this meta-analysis [12–19] (Fig. 1) (Table 1).

**Table 1:** Characteristics of included studies in meta-analysis

Study	Multicenter	Tumor location	Patient number (LRS)	Male patient (LRS)%	Patient number (ORS)	Male patient (ORS)%	Age (year, mean ± SD)	Resection type	TME	Neoadjuvant therapy
Braga 2007	No	Rectal	83	66	85	75	62.8 ± 12.6/65.3 ± 10.3	AR and APR	Yes	Yes
Ng 2008	No	Low 0–5 cm	51	61	48	63	63.7 ± 11.8/63.5 ± 12.6	APR	Yes	No
Ng 2009	No	Up	76	49	77	62	66.5 ± 11.9/65.7 ± 12.0	AR	No	Yes
Lujan 2009	No	Low	101	63	103	60	66.0 ± 9.9/67.8 ± 12.9	AR and APR	Yes	Yes
Liang 2011	No	NG	169	62	174	53	57.34 ± 14.13/57.36 ± 13.08	AR and APR	Yes	No
Bonjer 2015	Yes	Up mid low	699	52	345	53	66.8 ± 10.5/65.8 ± 10.9	AR and APR	(Upper) PME or TME	Yes
Green 2012	Yes	NG	253	56	128	54	69 ± (12)/69 ± (11)	AR and APR	77 % LRS 66 % ORS	Yes
Jeong 2014	No	Mid or low	170	65	170	65	59.1 ± (9.9)/57.8 ± (11.1)	AR and APR	Yes	Yes

*LRS* laparoscopic rectal surgery, *ORS* open rectal surgery, *AR* anterior resection, *APR* abdominoperineal excision, *TME* total mesorectal excision, *NE* neoadjuvant therapy, *NG* not given

**Fig. 2:**
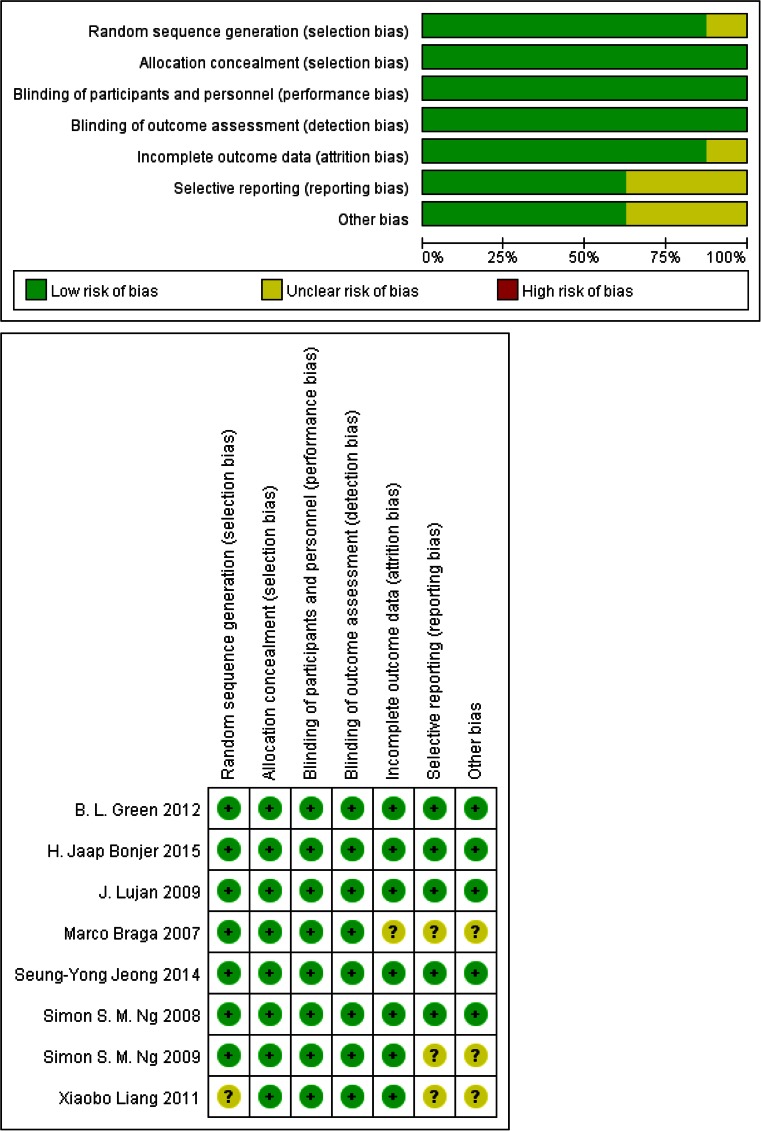
Quality assessment of included RCTs

The quality assessment of included RCTs was performed using Cochrane Collaboration’s tool. In these studies, blinding techniques were hardly feasible because of the different treatment procedures and the associated adverse effects. However, the review authors judge that the outcome is not likely to be influenced by lack of blinding. The risk of incomplete outcome data addressing, selective reporting, and other bias were not apparent across studies (Fig. 2).

### Meta-analysis results

#### Overall survival rate

**Fig. 3:**
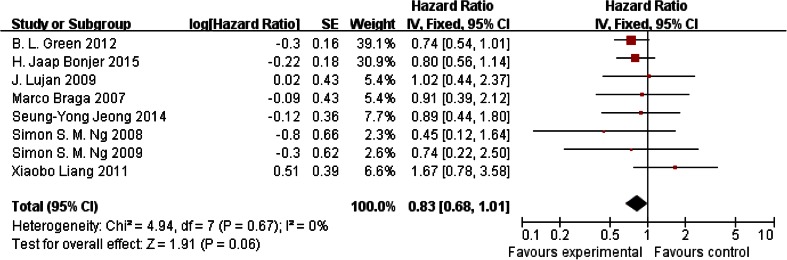
Overall survival rate reported in eight trials

Data for 3-year overall survival rate were reported in eight trials, and there was no heterogeneity among those trials (*P* = 0.06); thus, the fixed-effects model was used to pool the results. Data showed that no significant difference was found between the laparoscopy group and the open surgery group (HR = 0.83, 95 % CI [0.68, 1.01]; *P* = 0.06) (Fig. 3).

### Disease-free survival

**Fig. 4:**
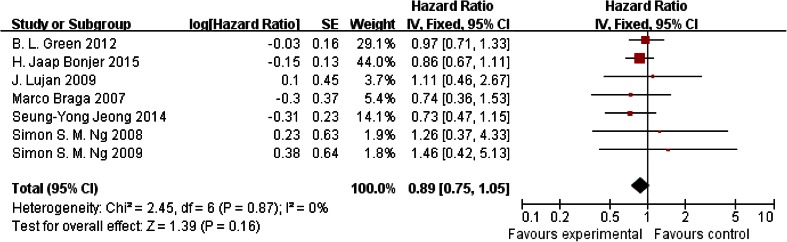
Disease-free survival rate reported in seven trials

Data for 3-year disease-free survival rate were reported in seven trials, and there was no heterogeneity among those trials (*P* = 0.16); thus, the fixed-effects model was used to pool the results. Data showed that no significant difference was found between the laparoscopy group and the open surgery group (HR = 0.89, 95 % CI [0.75, 1.05]; *P* = 0.16) (Fig. 4).

### The stage subgroup analysis

**Fig. 5:**
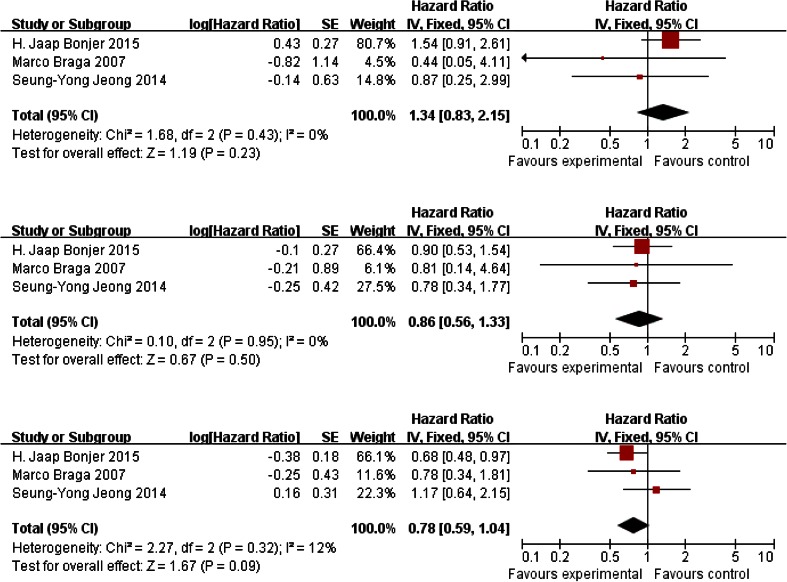
Disease-free survival rate in stages I, II, and III, reported in three trials

Three trials reported the data for 3-year disease-free survival rate in stages I, II, and III. The fixed-effects model was used to pool the results. Data showed that there was no significant heterogeneity in stage I (*P* = 0.23), stage II (*P* = 0.50), and stage III (*P* = 0.50). Only two trials reported the data for 3-year overall survival rate and the data was not enough for a meta-analysis (Fig. 5).

### Publication bias

The publication bias in this study was detected using a funnel plot of the meta-analysis result. The basic symmetry of the funnel plot suggested that there was no obvious publication bias in this meta-analysis. The Egger’s test for 3-year overall survival rates, 3-year disease-free survival rates, and the stage subgroup did not show any evidence of publication bias.

## Discussion

After the first report of laparoscopic colon resection in 1991 [20], laparoscopic surgery has progressively replace open colonic surgery in recent years for its short-term benefits and equivalent long-term survival [21]. But for rectal cancer, the evidence is still lacking for the oncological outcome between the two techniques. The golden standard to evaluate a technique for cure malignant cancer is whether the technique can adhere to the oncological standard of care. So, we preformed this meta-analysis to compare laparoscopic with open surgery for rectal cancer.

In the present meta-analysis, we examined the 3-year survival with the largest sample size using the time-to-event outcome and log hazard ratio to compare the two techniques for rectal cancer. As we know, time-to-event outcomes take account of whether an event takes place and also the time at which the event occurs, such that both the event and the timing of the event are important [22]. In survival analysis, the hazard ratio is the ratio of the hazard rates corresponding to the conditions described by two levels of an explanatory variable.

Two recent meta-analysis for long-term survival focused on relative risks showed a similar result for overall survival and disease-free survival (RR = 1.06, 95 % CI 0.96–1.18, *I* ^2^ = 14 %; RR = 1.04, 95 % CI 0.95–1.14, *I* ^2^ = 0 %) in 4 studies with 847 patients [23]. Though there are some other meta-analysis focus on this topic [24, 25], odds ratios and continuous variables were presented in the form of weighted mean differences with 95 % credible intervals. In systematic reviews and meta-analysis, time-to-event outcomes are most appropriately analyzed using hazard ratios. That is why we use hazard ratio instead of odds ratio to analyze the data.

Based on the large number of patients, we also preformed a subgroup analysis by stage to investigate whether patients could have better disease-free survival after laparoscopic surgery. Interestingly, in Bonjer’s study [12], disease-free survival for laparoscopic surgery in stage III subgroup is better than that for open surgery. But this result is not supported when looking at aggregate stage data. With the activating of COLOR II trial and ACOSOG Z6051 trial, more results will be published to make it possible to analyze overall survival and disease-free survival at 5 years.

Another point which needs attention is the high conversion rate. Laparoscopic surgery for rectal cancer is otherwise a rather complicated surgery than other laparoscopic colectomy because the surgical fields for rectal surgery are confined by the narrow and deep pelvis, and total mesorectal excision and autonomic nerve preservation are prerequisites for functional and oncological safety [26–28].

Some limitations of this meta-analysis should also be acknowledged when interpreting the results: First, neoadjuvant chemotherapy and chemotherapy after surgery are quite important factors which may play a role on the results [29], since different chemotherapy strategies for laparoscopic and open surgery would have unpredictable influence on the long-time outcomes. Second, we failed to detect the relationship between different locations of tumor and the 3-year survival due to lack of information. The specific location may affect the surgery approach which may bring about clinical heterogeneity between trials. The surgical skill varied from different surgeons, which may also have some potential effects on the outcomes.

In a word, it is clear that laparoscopic surgery has better short-time outcomes compared with open surgery through previous research [5–7, 23]. While in this analysis which is based on the 3-year follow-up outcomes, we concluded that there was no significant difference between the two techniques in terms of 3-year survival, and they are both safe and effective.

## References

[1] Ferlay J, Soerjomataram I and Ervik M, et al (2013) GLOBOCAN 2012 v1. 0. Cancer incidence and mortality worldwide: IARC CancerBase

[2] Siegel R, Ma J, Zou Z, Jemal A (2014). Cancer statistics, 2014. CA: Cancer J Clin.

[3] Chen W, Zheng R, Zhang S (2014). Annual report on status of cancer in China, 2010. Chin J Cancer Res.

[4] American CS: Cancer facts & figures. The Society, 2014

[5] van der Pas MH, Haglind E, Cuesta MA (2013). Laparoscopic versus open surgery for rectal cancer (COLOR II): short-term outcomes of a randomised, phase 3 trial. Lancet Oncol.

[6] Kang S, Park JW, Jeong S (2010). Open versus laparoscopic surgery for mid or low rectal cancer after neoadjuvant chemoradiotherapy (COREAN trial): short-term outcomes of an open-label randomised controlled trial. Lancet Oncol.

[7] Guillou PJ, Quirke P, Thorpe H (2005). Short-term endpoints of conventional versus laparoscopic-assisted surgery in patients with colorectal cancer (MRC CLASICC trial): multicentre, randomised controlled trial. Lancet.

[8] National CCN: NCCN Guidelines®: Colon/Rectal Cancer., 2015

[9] Higgins J, Altman DG, Gøtzsche PC et al (2011) The Cochrane Collaboration’s tool for assessing risk of bias in randomised trials. BMJ 343 10.1136/bmj.d5928 PMC3196245 22008217

[10] Green S (2011) Cochrane handbook for systematic reviews of interventions version 5.1. 0 [updated March 2011]. The Cochrane Collaboration

[11] DerSimonian R, Laird N (1986). Meta-analysis in clinical trials. Control Clin Trials.

[12] Bonjer HJ, Deijen CL, Abis GA (2015). A randomized trial of laparoscopic versus open surgery for rectal cancer. N Engl J Med.

[13] Liang X, Hou S, Liu H (2011). Effectiveness and safety of laparoscopic resection versus open surgery in patients with rectal cancer: a randomized, controlled trial from China. J Laparoendosc Adv Surg Tech A.

[14] Braga M, Frasson M, Vignali A, Zuliani W, Capretti G, Di Carlo V (2007). Laparoscopic resection in rectal cancer patients: outcome and cost-benefit analysis. Dis Colon Rectum.

[15] Ng SS, Leung KL, Lee JF (2008). Laparoscopic-assisted versus open abdominoperineal resection for low rectal cancer: a prospective randomized trial. Ann Surg Oncol.

[16] Green BL, Marshall HC, Collinson F (2013). Long-term follow-up of the Medical Research Council CLASICC trial of conventional versus laparoscopically assisted resection in colorectal cancer. Br J Surg.

[17] Ng SS, Leung KL, Lee JF, Yiu RY, Li JC, Hon SS (2009). Long-term morbidity and oncologic outcomes of laparoscopic-assisted anterior resection for upper rectal cancer: ten-year results of a prospective, randomized trial. Dis Colon Rectum.

[18] Jeong SY, Park JW, Nam BH (2014). Open versus laparoscopic surgery for mid-rectal or low-rectal cancer after neoadjuvant chemoradiotherapy (COREAN trial): survival outcomes of an open-label, non-inferiority, randomised controlled trial. Lancet Oncol.

[19] Lujan J, Valero G, Hernandez Q, Sanchez A, Frutos MD, Parrilla P (2009). Randomized clinical trial comparing laparoscopic and open surgery in patients with rectal cancer. Br J Surg.

[20] Jacobs M, Verdeja JC, Goldstein HS (1991). Minimally invasive colon resection (laparoscopic colectomy). Surg Laparosc Endosc.

[21] Theophilus M, Platell C, Spilsbury K (2014). Long-term survival following laparoscopic and open colectomy for colon cancer: a meta-analysis of randomized controlled trials. Colorectal Dis.

[22] Tierney JF, Stewart LA, Ghersi D, Burdett S, Sydes MR (2007). Practical methods for incorporating summary time-to-event data into meta-analysis. Trials.

[23] Zhang FW, Zhou ZY, Wang HL (2014). Laparoscopic versus open surgery for rectal cancer: a systematic review and meta-analysis of randomized controlled trials. Asian Pac J Cancer Prev.

[24] Jiang JB, Jiang K, Dai Y (2015). Laparoscopic versus open surgery for mid-low rectal cancer: a systematic review and meta-analysis on short- and long-term outcomes. J Gastrointest Surg.

[25] Trastulli S, Cirocchi R, Listorti C (2012). Laparoscopic vs open resection for rectal cancer: a meta-analysis of randomized clinical trials. Colorectal Dis.

[26] Bege T, Lelong B, Esterni B (2010). The learning curve for the laparoscopic approach to conservative mesorectal excision for rectal cancer: lessons drawn from a single institution’s experience. Ann Surg.

[27] Kayano H, Okuda J, Tanaka K, Kondo K, Tanigawa N (2011). Evaluation of the learning curve in laparoscopic low anterior resection for rectal cancer. Surg Endosc.

[28] Son GM, Kim JG, Lee JC (2010). Multidimensional analysis of the learning curve for laparoscopic rectal cancer surgery. J Laparoendosc Adv Surg Tech A.

[29] Sauer R, Becker H, Hohenberger W (2004). Preoperative versus postoperative chemoradiotherapy for rectal cancer. N Engl J Med.

